# Supplementary material for: A Humanized Yeast Phenomic Model of Deoxycytidine Kinase to Predict Genetic Buffering of Nucleoside Analog Cytotoxicity
Source: Genes (Basel). 2019 Sep 30;10(10):770. doi: 10.3390/genes10100770 (PMC6826991; doi:10.3390/genes10100770)
Supplement: Supplementary file 1 [file genes-10-00770-s001.zip › Additional_Files/Additional_File7_GOTermHeatmaps.zip!/Additional_File7_GOTermHeatmaps/ATP-dependent_RNA_helicase_activity.pdf]

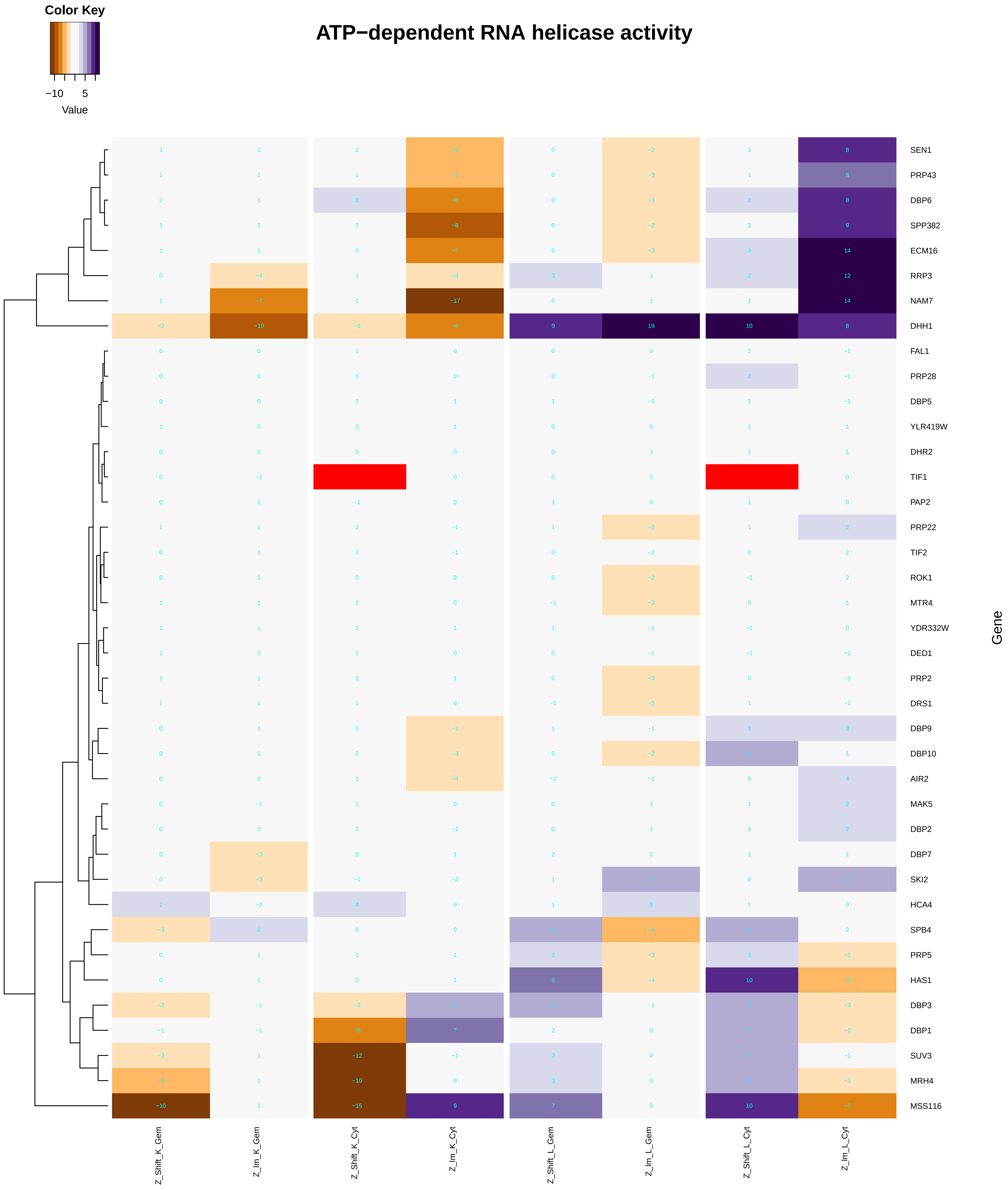

ATP−dependent RNA helicase activity
Color Key
−10
5
Value
SEN1
PRP43
DBP6
SPP382
ECM16
RRP3
NAM7
DHH1
FAL1
PRP28
DBP5
YLR419W
DHR2
TIF1
PAP2
PRP22
TIF2
ROK1
MTR4
YDR332W
DED1
PRP2
DRS1
DBP9
DBP10
AIR2
MAK5
DBP2
DBP7
SKI2
HCA4
SPB4
PRP5
HAS1
DBP3
DBP1
SUV3
MRH4
MSS116
Gene
Z_Shift_K_Gem
Z_Im_K_Gem
Z_Shift_K_Cyt
Z_Im_K_Cyt
Z_Shift_L_Gem
Z_Im_L_Gem
Z_Shift_L_Cyt
Z_Im_L_Cyt

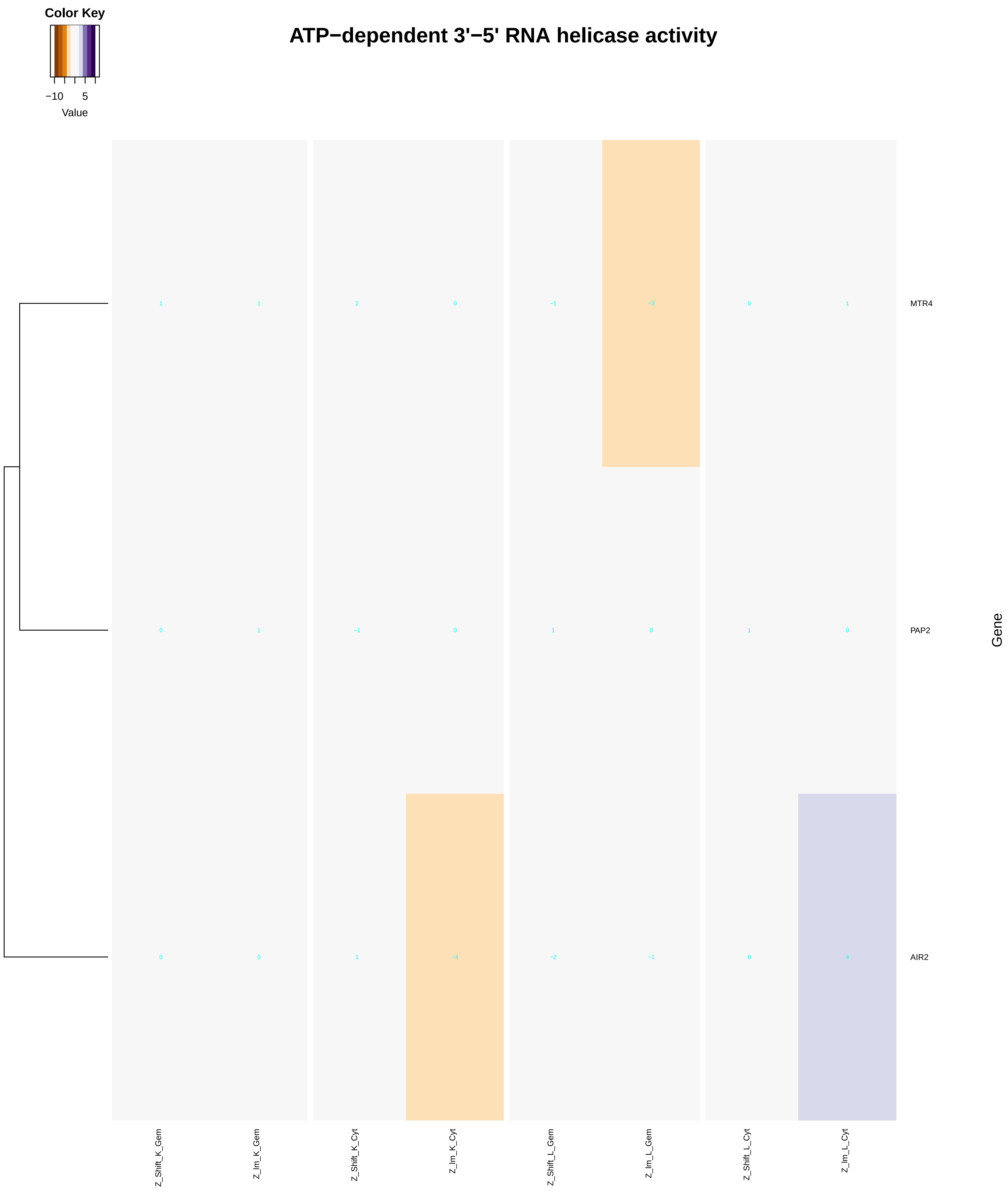
Color Key
−10
5
Value
ATP−dependent 3'−5' RNA helicase activity
MTR4
PAP2
AIR2
Gene
Z_Shift_K_Gem
Z_Im_K_Gem
Z_Shift_K_Cyt
Z_Im_K_Cyt
Z_Shift_L_Gem
Z_Im_L_Gem
Z_Shift_L_Cyt
Z_Im_L_Cyt
